# Medical students’ experiences in learning to perform pelvic examinations: a mixed-methods study

**DOI:** 10.5116/ijme.617f.b261

**Published:** 2021-11-26

**Authors:** Johanna Danielsson, Cecilia Hadding, Martin Fahlström, Ulrika Ottander, David Lindquist

**Affiliations:** 1 Department of Clinical Sciences, Professional Development, Umeå University, Umeå, Sweden; 2 Department of Clinical Sciences, Obstetrics and Gynecology, Umeå University, Umeå, Sweden

**Keywords:** Gender differences, pelvic examination, learning opportunities, medical students, obstetrics and gynaecology clerkship

## Abstract

**Objectives:**

We aimed to explore learning experiences among medical students learning to perform pelvic examinations and to identify factors that facilitate their training.

**Methods:**

A mixed-methods study including a web-based survey and focus group discussions (FGDs) was conducted among medical students who had completed their obstetrics and gynaecology (ObGyn) clerkship. The FGDs were recorded, transcribed and analysed using qualitative content analysis with systematic text condensation. Survey factors were compared using the χ^2^ test or Fisher's exact test.

**Results:**

160 students (97 female, 61 male, two other) at six universities in Sweden responded to the survey. Two mixed FGDs were conducted. The majority (87%) of the students experienced confidence in performing pelvic examinations, stating that sufficient, repeated training opportunities and support from a clinical tutor were crucial components of the learning experience. Prior to the ObGyn clerkship, negative expectations were more common among male students. The male participants experienced having a disadvantage because of their gender, while female students considered their gender an advantage (p<0.001, N=121, Fisher's Exact Test). The clinical tutor and the use of professional patients (PPs) had a fundamental role in providing learning opportunities by including the student in patient care activities.

**Conclusions:**

The importance of the clinical tutor, as well as the use of PPs, are important factors when planning education in pelvic examinations, and this knowledge could be used when educating other intimate examinations during medical school. In addition, similar investigations on students' experience in training other intimate examinations could be considered.

## Introduction

Some clinical specialities are more male- or female-dominant than others. In 2018, in the USA, 83% of the residents in obstetrics and gynaecology (ObGyn) were female.^1^ Too little gender diversity is not desirable since it might result in teams that are too homogenous.^2^ Multifaceted health care teams are highly valued since this heterogeneity enhances problem-solving.^3^ Considering that physicians may choose their speciality based on their medical school experience, the importance of the student clerkship cannot be stressed enough. In particular, the ObGyn clerkship has been targeted as critical in terms of recruiting future residents to the speciality.^1^^,^^4^^-^^6^Research has shown that male students experience their gender as a barrier in ObGyn training and that this may increase the number of male students who choose not to pursue careers within the field.^6^^,^^7^ The feeling of having a disadvantage because of one's male gender may be due to being denied consent to participate in clinical care to a greater degree than female students. Gender bias among faculty members is another identified reason.^4^^-^^6^ Taken together, these factors may result in male students receiving less training in performing ObGyn examinations and clinical evaluations.Not all medical graduates will enter ObGyn residencies, but the majority of medical doctors will provide care to female patients regardless of their chosen speciality. All physicians should be confident in when to refer a patient and how to interpret patients' health issues, and be able to understand a written statement by a specialist in ObGyn. Therefore, it is important that all medical students gain adequate clinical experience and competence in women's health.

The curricula for medical students vary due to during which semester obstetrics and gynaecology is taught, as well as how students gain knowledge and experience in performing pelvic examinations during their clinical clerkships. In some medical schools, the use of professional patients (PP) is a well-established part of medical education. A PP is a female patient who voluntarily undergoes vaginal examinations and who is trained to provide feedback to students on their communication and technical skills. The use of PP, as well as women's experiences of pelvic examinations, have been studied previously in Sweden,^8^^-^^10^ but less is known about the medical students' experiences and what factors facilitate adequate training.

The objective of this study was to determine whether medical students in Sweden experience that they have acquired enough knowledge in their ObGyn clerkship to feel confident in performing pelvic examinations (speculum and/or vaginal examinations) and to detect whether there are differences in the experiences of medical students with respect to gender.

The main research questions were:

    ·      Do medical students experience that they have acquired adequate training in and knowledge of pelvic examinations during their ObGyn clerkship?

    ·      How do medical students experience their learning opportunities during their ObGyn clerkship?

    ·      Do medical students experience that their gender influences their ObGyn clerkship?

    ·      What factors are important for students when learning pelvic examinations?

## Methods

### Study design and participants

Both quantitative and qualitative methods were used to fulfil the aims of the study. By describing the relations between the qualitative and quantitative findings (complementarity), understanding a phenomenon can be facilitated and improved.^8^^,^^9^

Ethical principles were followed in all parts of the project according to the Helsinki declaration. The participants are not identifiable, and the data are presented at the group level only. No sensitive personal data were collected. The study does not deal with personal data on offences and is not associated with any physical or mental intervention. For these reasons, the design of the study does not fall under the Ethical Review Act of research in Swedish law (2003:460). An ethical regulatory approval request was therefore not applied for. Data were collected from July 2nd to November 11th, 2020. The survey was submitted to students who had finished their ObGyn clerkship at Umeå University, Lund University, Karolinska Institutet, Uppsala University and Linköping University. The students were approached through social media groups, e-mail and university platforms with information regarding the study and the survey. Participation was voluntary and anonymous.

The qualitative part of the study was conducted through two focus group discussions (FGDs). The medical students who participated in the FGDs (N=10) had finished most of their ObGyn clerkship at Umeå University in November 2020.^13^^,^^14^ Each group consisted of five participants. The groups were mixed regarding gender but homogenous with respect to the site of clerkship and age. Six male and four female students aged 25-28 years accepted the invitation to participate in a FGD.

Participants in the FGDs were recruited through social media. When four participants had been recruited, chain-referral sampling was used for further recruitment. Potential participants were approached with the same information regarding the study as those who participated in the quantitative part of the study and consented to participate. Initially, a convenience sampling technique was used to recruit the students. This technique has been shown to work well when the studied population is predefined.^15^ Since the medical students were harder to reach than expected, the chain-referral sampling technique was later applied, with participants serving as referral sources.^16^ Participation was voluntary, and the identities of the participants were later coded in such way that the personal information was deleted. The only information saved about the participants was their gender identification and age.

### Data collection procedures

The survey was based on one used in a previous study and was adapted to local conditions.^4^ Content validity was addressed in two ways based on recommendations published by Polite and colleagues. ^17^ First, a pilot study was performed and evaluated in a sample of five medical doctors. Minor linguistic adjustments were made, and the time needed to answer all questions was estimated to be 10 minutes. Second, three of the authors, who are senior lecturers with long experience in medical education (MF, UO, DL), reviewed the questions and came to an agreement on the relevance of including each question.

The survey consisted of 52 questions. The majority of the questions were answered using a 4-point Likert scale (never, occasionally, often, always). A four-grade semi-quantitative scale was used to avoid having a neutral mid-point alternative.^17^ Only one answer could be submitted per question. The first part of the survey consisted of four demographic questions that addressed what gender the students identified themselves with, their year of birth, their country of birth, and at which university they participated in their ObGyn clerkship.

The second part of the survey concerned the clinical rotation in gynaecology. It included 25 questions. One 4-point Likert question investigated the experience the student had prior to the ObGyn clerkship in terms of performing pelvic examinations. There were four questions regarding obtaining consent to participate in different clinical situations and on the number of hands-on procedures the student had been allowed to participate in and/or to perform. There were seven 4-point Likert questions. The participants were asked to estimate the number of times they were not granted permission to collect a patient's history and observe or perform examinations. Students were asked to estimate the number of examinations they performed/were not allowed to perform. One question concerned whether the student felt that gender had influenced the clerkship experience ("Yes, in a positive way", "Yes, in a negative way" or "No"). The third part of the survey concerned the obstetrics part of the clerkship. It consisted of 18 questions designed in the same way as those in the second part but adapted to the obstetrics rotation. The fourth part of the survey consisted of six questions. It included questions regarding the students' overall experience of the ObGyn clerkship and the likelihood of the student considering an ObGyn residency in the future. One question aimed to ascertain whether the student would find it valuable to approach the subject of pelvic examinations and their related expectations and misgivings in the preclinical part of their medical training. The survey included questions concerning incidents of feeling discriminated against or harassed during the clerkship.

Two FGD web sessions were conducted. The moderator (JD) called for stories about how the students had experienced their clinical clerkship, and an observer (CH) evaluated the interaction during and atmosphere of the discussion. A semi-structured interview guide consisting of open-ended questions was used; it emphasised descriptive accounts of events ("how" and "when").^13^ The interviews were recorded in audio files.

### Data analysis

Responses from the survey were converted into numerical values and analysed using uni- and bivariate analyses. Comparisons were performed using χ^2^ tests for categorical data, and Fisher's exact test was used when the conditions were not met for a valid χ^2^ test. The variable frequencies never/occasionally and often/always were combined where appropriate to enable analysis. In some cases, the variables were combined in another manner to detect when a student never or always experienced a specific phenomenon. For statistical significance, a p-value less than 0.05 was used. Cronbach's alpha was used to test the reliability of the test scores by including sets of closely related items. Quantitative statistical analysis was performed using IBM SPSS statistics version 27.

### Qualitative content analysis with systematic text condensation

The focus group data were transcribed, and the participants were given an identification number (10 to 19). The transcripts were analysed via conventional content analysis with systematic text condensation.^14^^,^^15^ All transcription of the data was performed manually without the use of a database. In the first step of the analysis, initial themes were identified by repeatedly reading through the transcript. Thereafter, the transcript was read verbatim in a more systematic manner in order to identify meaning units. A meaning unit is a segment of text that answers the main questions of the study or contains information that may offer important insight for the study. The meaning units were identified without the use of an already defined template. The units sampled were those that concerned how the student had experienced their ObGyn clerkship and their learning opportunities. The units were sorted into code groups. Since a conventional approach to qualitative content analysis was used, the code groups were created during the analytical process and had not been described prior to the study.^19^ The phenomena comprising one code group were split to form one corresponding subgroup each. Thus, each subgroup described only one phenomenon. This was validated through the condensation of the subgroups. In the last part of the analytical process, the condensate was rewritten into an analytical text describing the concepts and phenomena.^18^

## Results

### Survey results

One hundred and sixty students participated, of whom 97 (61%) were female, 61 (38%) were male, and 2 (1%) identified as "other". All surveys were complete, with no missing data. The mean age of the respondents was 27.8 years (range: 23-48 years). The majority of the respondents, 147 (92%), were born in Sweden. A total of 13 students were born outside of Sweden, nine (5.5%) of whom were born in another Nordic country. Only four (2.5%) students were born outside the Nordic countries. The respondents were students at six different universities in Sweden (Table 1). The students attended their ObGyn clerkship in 2019 and 2020, 17(11%) and 143 (89%), respectively.

**Table 1:** Student distribution and universities represented

University	Frequency	Percentage
Karolinska Institutet	26	**16**
Linköping University	2	**1**
Lund University	55	**34**
Gothenburg University	27	**17**
Umeå University	46	**29**
Uppsala University	4	**3**
Total	160	**100**

A majority of the participants, 136 (85%), had no experience performing pelvic examinations (speculum and/or vaginal examinations) prior to their ObGyn clerkship. Those with previous experience did not report being more comfortable performing pelvic examinations at the end of the clerkship than those who did not have any experience prior to their gynaecological rotation.

When asked about the impact of the ObGyn rotation, 122 (76%) participants stated that their interest in the speciality had increased, seven (4%) stated that their interest in the field had decreased, and 31 (19%) stated that their clinical clerkship had not influenced them. Just over half of the respondents (88; 55%) claimed that they were considering a future as a resident in ObGyn, 30 (19%) students stated that they felt unsure about doing so, and 42 (26%) participants indicated that they were not considering a future in ObGyn.

When asked about the possible need to prepare students prior to the clinical rotation, 45 (28%) participants stated that preparation would have been valuable. However, 26 (16%) felt unsure about this value, and 89 (56%) said they did not see any value in discussing thoughts and feelings regarding the ObGyn clerkship during the preclinical period. There was no difference concerning gender.

The majority of the students, 139 (87%), reported no experience of being discriminated against during the clerkship. The mean number of examinations performed was 10.35 ± 4.63. In total, 139 (87%) students stated that they felt confident performing a pelvic examination at the end of their clinical clerkship (Table 2).

**Table 2:** Gynaecology rotation experiences reported by the students (N = 160)

Experiences	Never/ Occasionally (%)	Sometimes/ Always (%)
Experienced being encouraged to participate in the clinical work	29 (18)	131 (82)
Experienced not being allowed to participate in a consultation (by the patient)	157 (98)	3 (2)
Experienced not being allowed to collect patient history (by the patient)	159 (99)	1 (1)
Experienced not being allowed to observe an examination (by the patient)	157 (98)	3 (2)
Experienced not being allowed permission to perform an examination (by the patient)	153 (96)	7 (4)
Experienced not being allowed permission to participate (by supervisor)	159 (99)	1 (1)
Experienced being supported when performing gynaecological examinations (by supervisor)	6 (4)	154 (96)
Experienced feeling included as part of the team (by healthcare professionals)	28 (18)	132 (83)
Experienced being confident in performing gynaecological examinations (speculum and/ or vaginal examination) at the end of the Ob/Gyn clerkship	21 (13)	139 (87)
Experienced feeling discriminated against during the gynaecological placement		
By midwife	156 (98)	4 (3)
By doctor	149 (93)	11 (7)
By auxiliary nurse	158 (99)	2 (1)

Test-score reliability was measured using Cronbach's alpha grouping closely related 4-point Likert questions in groups of four. The Cronbach's alpha coefficient ranged between 0.68-0.83 for the different groups.

### Gender differences in gynaecology clerkship experiences

In total, 97 (61%) female and 61 (38%) male students answered the survey; two (1%) students identified as neither female nor male and were therefore excluded from the analysis considering gender differences. The same proportion (82%); (n=80 and 50 respectively) of female and male students stated feeling encouraged to participate during the gynaecology rotation. There was no significant difference with respect to gender in the number of examinations observed or performed during the clerkship experience (Table 3).

**Table 3:** Gender differences in observed and performed examinations

Examinations	Female students (%) (N = 97)	Male students (%) (N = 61)	p-value^*^
Number of examinations observed			
< 10	23 (24)	9 (15)	0.173
≥ 10	74 (76)	52 (85)	0.173
< 15	40 (41)	28 (46)	0.564
≥ 15	57 (59)	33 (54)	0.564
Number of examinations performed			
< 10	55 (57)	38 (62)	0.487
≥ 10	42 (43)	23 (38)	0.487
< 15	76 (78)	51 (84)	0.418
≥ 15	21 (22)	10 (16)	0.418

^*^It is based on the χ^2^ test

When students were asked about ever being denied permission to participate in consultations, a significant difference was observed, with 79 (81%) female and 12 (20%) male students reporting never experiencing being denied permission to participate in clinical consultations (χ^2^ (1,N =158)= 58.5 p=0.001) and 94 (97%) female and 44 (72%) male students reporting never being denied permission to collect patient history (p = 0.001, N=158, Fisher's Exact Test). Similarly, significant differences were observed in experiences of being denied permission to observe or perform pelvic examinations (Table 4).

Overall, 79 (81%) female students and 42 (69%) male students perceived that their gender influenced the gynaecological clerkship. Of these, all the female students stated that their gender had a positive impact on their clerkship experience, while 41/42 (98%) of the male students stated that their gender had a negative influence on their clerkship experience (p <0.001, N=121, Fisher's Exact Test).

There was no significant difference with respect to gender (n=82; 85% female vs n=48; 79% male) in terms of experiencing being included by the clinical tutor as a part of the team or in terms of experiencing discrimination or harassment by a doctor (6% female vs 8% male), midwife, or auxiliary nurse (11% female vs 11% male).

**Table 4:** Differences in the number of times permission to observe and perform examinations were denied by gender

Number of times	Female students (%) (N = 97)	Male students (%) (N = 61)	p-value^*^
Number of times permission to observe a gynaecological examination was denied			
0	96 (99)	27 (44)	< 0.001
≥ 1	1 (1)	34 (56)	< 0.001
<3	97 (100)	37 (61)	< 0.001^**^
≥3	0 (0)	24 (39)	< 0.001^**^
≤5	97 (100)	52 (85)	< 0.001^**^
>5	0 (0)	9 (15)	< 0.001^**^
Number of times permission to perform an examination was denied			
0	66 (68)	11 (18)	< 0.001
≥1	31 (32)	50 (82)	< 0.001
<3	91 (94)	41 (67)	< 0.001
≥3	6 (6)	20 (33)	< 0.001
≤5	96 (99)	50 (82)	< 0.001^**^
>5	1 (1)	11 (18)	< 0.001^**^

*it is based on the χ2 test

**It is based on Fisher's exact test

### Gender differences in the obstetric clerkship experience

There was no difference with respect to gender in feeling encouraged to participate in the delivery ward. However, more men, 11 (18%) than women, 4 (4%) stated that they never experienced being encouraged to participate in the delivery ward (p=0.005, N=158, Fisher's Exact Test). A majority of female students, 90 (93%) and 31 (51%) male students, stated that they were always allowed to participate during childbirth (p<0.001, N=127, Fisher's Exact Test).

One (1%) female and 5 (8%) male students stated that they were always denied permission to perform vaginal examinations in the delivery ward (p=0.032, N=158, Fisher's Exact Test).

Of the students who experienced that their gender influenced their obstetric clerkship experience, 67 were female, and 29 were male (χ^2^(1, N=158)=7.28 p=0.007), of which the female students stated that their gender had influenced their clerkship experience in a positive way, while the male students experienced that their gender had affected their clerkship experience negatively (χ^2^(1,N=96)=96.0,p<0.001). There was no difference with respect to gender in experiences of being discriminated against or harassed by an auxiliary nurse, midwife or doctor (range 0-14%).

### Gender differences in the ObGyn clerkship in general

At the end of the clerkship, 130 (82%) students stated that their interest in obstetrics and gynaecology had been influenced by the clerkship experience. A majority, with no significant difference with respect to gender, experienced that their interest in the field had increased. Eighty-eight (91%) female and 49 (80%) male students stated that they felt confident in performing examinations at the end of the ObGyn clerkship. Only 2 (2%) female and 2 (3%) male students stated that they never felt comfortable performing an examination, even at the end of the clinical clerkship.

### Results from the focus group discussions

In the FGDs, engaging stories about how the students experienced their ObGyn clerkship experiences were told. The discussion provided information about five areas of interest, divided into 14 different phenomena that concerned how the students had experienced their learning opportunities and their ObGyn clerkship in general. The areas of interest are presented as code groups. Each code group was divided into 2-4 subgroups (Table 5).

**Table 5:** Division of code and subgroups

Code group	Subgroup
Lessons learned	Learning the importance of soft values
	Feeling more comfortable with performing examinations
	Confidence in performing examinations after considerable practice
Exceeded expectations and refuted prejudices	Notions of patients
	Stories and misgivings
The structure of the clerkship	Missing the opportunity to discuss a case without the patient being present
	Professional patients: real people in a comfortable setting
Involving the student: an effective teaching tool	The introduction: being introduced as a future colleague
	Being recognised makes a difference
	Supervisors that educate
	Adjust the teaching to the students' knowledge level
Patient autonomy and being denied permission	Understanding that you may not be allowed to participate
	Easier to accept being denied permission when given a reason why
	Anxiety about restricting patient autonomy

### Lessons learned

#### Learning the importance of soft values

There was a focus on soft values during the clerkship, and much emphasis was placed on patient integrity. The focus on respecting the naked body was not something the students recognised from other clinical rotations. The participants expressed that it would be valuable if health professionals within all fields of medicine also focused on respecting patient integrity and said that they were sometimes appalled by their failure to do so. The participants stated that now that they had completed their ObGyn clerkship, they would apply the focus on soft values to their treatment of patients within all fields of medicine in the future.

"This you will carry with you throughout your education; we collect pieces throughout our education in order to develop our technique in how to treat patients. In this, the ObGyn clerkship has really contributed." - Male, 28.

#### Feeling more comfortable with performing examinations

The participants said that the education and knowledge that they had acquired in the ObGyn clerkship experience had made them more comfortable with performing intimate and invasive examinations in general. Students participating in the FGD claimed that they now felt more comfortable performing a rectal examination or a proctosigmoidoscopy since the gynaecology clerkship had equipped them with both experience and knowledge regarding how to handle examinations where the patient may feel vulnerable or uncomfortable.

"I haven't performed as many proctosigmoidoscopies as I have gynaecological examinations, but I will feel more comfortable performing one now … I have acquired knowledge on how to care for patients in vulnerable situations." - Male, 28.

#### Confidence in performing examinations after considerable practice

There was consensus among the participants that they felt prepared to perform pelvic examinations at the end of the clerkship and were comfortable doing so. They said that when learning to perform an examination, it is valuable to practice many times, as this allows one to assess what is normal and what is not. They expressed that they had been offered more opportunities to practice their examination technique than is typical in other fields of medicine.

"I think we have been able to perform more examinations in the ObGyn clerkship than in most other clinical rotations. I would claim that I'm better at performing a pelvic examination than I am in assessing the prostate gland, just because I have been able to attend so many shifts during ObGyn." - Male, 26.

### Exceeded expectations and refuted prejudices

#### Notions of patients

Some had expected to be denied permission to examine to a much greater extent, but instead, they had been able to perform more examinations than they expected. There was a notion that younger patients would be more unwilling to let a student examine them. Therefore, the students had been pleasantly surprised and did not observe any difference with respect to patient age in whether they were granted permission to participate or to examine patients. The students stated that their expectations of the ObGyn clerkship had been exceeded in a positive way.

"I had expected that the older women would be more open to letting me examine and that the younger women would find the situation harder and therefore would not let me examine. But I have not experienced any difference regarding patient age and being allowed to perform examinations." - Female, 26.

#### Stories and misgivings

Some participants claimed that they, to a large extent, had based their expectations of the clerkship on misgivings and stories from senior students who had experienced being denied permission, especially those who had been denied permission to participate because of their male gender. It was proposed by the students that the misgivings and stories that the male students based their expectations on may have emerged because their fellow students tended to share negative experiences rather than positive experiences.

"I had heard from several people that many male students have not been allowed to participate during examinations and patient consultations because of their gender. I was expecting to not be granted permission to participate in every other visit, but that has not been the case, and thus, my expectations have been exceeded." - Male, 25.

### The structure of the clerkship

#### Missing the opportunity to discuss a case without the patient being present

The students experienced that the opportunity to discuss and practice diagnostics was to some extent lost during the clerkship. They claimed that since both the patient and the clinical tutor were always present during the consultation and examination, they were not given the opportunity to express their thoughts or suggestions regarding the patients' condition with their clinical tutors without the patient being present within hearing distance.

"I think it has been hard to learn the diagnostics in gynaecology since there has not been enough time to air my thoughts or suggestions since the patient is always present." - Female, 26.

#### Professional patients: real people in a comfortable setting

Students that had the opportunity to practice examination techniques on a professional patient strongly appreciated that. They experienced that it was valuable to perform their first actual examination on a person who was able to give them instant feedback. The experience made them feel less insecure and nervous about performing pelvic examinations on real patients, even though the situation would be different.

"The situation is different when you get out in the clinic, and if you have been able to perform an examination on a real patient in a comfortable setting before this, a patient who instructs you and helps you, it gets much easier." - Male, 25.

### Involving the student: an effective teaching tool

#### The introduction: being introduced as a future colleague

The participants highlighted the relationship between how they were introduced by their clinical tutor and being allowed permission to participate. They expressed that it might be positive to introduce the student as a future colleague or as an almost qualified doctor to make the patient feel more comfortable with the students as professionals during the visit. Additionally, this would give the patient a feeling that the student has experience and competence in managing patients.

"It might be better to use the phrase future colleague in this sort of situation where it means a lot that the patient feels safe and comfortable. And that they have confidence in their caregiver. And I think that has been good in this clinical rotation." - Female, 28.

#### Being recognised makes a difference

The participants mentioned a good climate among the health care professionals during the ObGyn clerkship. They talked of health care professionals who recognised their presence, not by expressing dissatisfaction but by greeting the students and including them in their clinical duties. The participants experienced that they had better chances of being allowed by patients to participate if the supervisors included them in the patient care early in the process. They claimed that early involvement made patients more comfortable with having the student present and that the student, on the other hand, felt more comfortable with being present.

"Just to have someone say hey, you are a student, and you are with me, who introduces you to the patient and who spends some time and energy on you. That's enough. We don't need more than that." - Female, 25.

#### Supervisors that educate

Students participating in the FGDs experienced that the quality of the supervisors was very high. They felt encouraged to perform many examinations, and they were provided with opportunities to discuss differential diagnoses, patients' conditions and treatments. There was a preference for supervisors who continually explained what they were doing when examining and who included the student in decisions. This also made the student feel more a part of the team. One student expressed that the more engaged the supervisor was with him, the more involved he was in learning.

"It's so valuable to be given time to ask questions, to be able to participate and to have a supervisor who remains aware that he or she is supposed to teach and instruct you so that I don't have to feel like a passive shadow." - Male, 26.

#### Adjust the teaching to the students' knowledge level

Though the quality of clinical tutors, in general, was high, one student expressed that some of the more senior gynaecologists had a difficult time understanding the competence level of the students. This resulted in descriptions and reasoning far above what the student understood. The most valuable information that supervisors provided was based on their years of experience and included basic tips that could not be found in the course literature regarding how to improve the experience for the patient during the examination or solid tips on how to improve one's examination technique.

"My experience is that it is better to keep the supervising and teaching at a more basic level and to teach us concrete tips that we can apply in the clinic, such as how to better angle the speculum so that it doesn't come in contact with the urethra when examining." - Male, 26.

### Patient autonomy and being denied permission

#### Understanding that you may not be allowed to participate

The students expressed that they were aware of the fact that they could be denied permission to participate in observing and performing examinations during the ObGyn clerkship. They said it was perfectly reasonable that some patients do not want students present, especially students of the opposite gender when being examined. Some participants claimed that it even felt natural not to participate in some instances, for example, when the patient was from a culture where it is not appropriate to be intimately examined by a male doctor or when the patient was a victim of sexual assault.

"I was quite prepared that there was a risk of being denied permission to participate, and I think it's perfectly reasonable. So, I did understand that I was not allowed to participate at the time, but of course, it's much nicer to feel welcome." - Male, 25.

#### Easier to accept being denied permission when given a reason why

Sometimes it was harder to accept being denied permission to participate when no reason was given regarding why the patient did not want the student to participate in her care. This may lead to students losing self-confidence. Stress can accumulate since there is a fear about not being granted permission to see what one is supposed to see and do what one is supposed to do during the clerkship.

"If you are not allowed to participate, you start to wonder… You want an explanation even if you don't need one. It's hard not to think about it." - Male, 26.

#### Anxiety about restricting patient autonomy

Male students sometimes felt that they put patients in a difficult situation when asking them for consent to participate during their appointment. They described a feeling of restricting the autonomy of the patient. There was a feeling of anxiety expressed as having a knot in one's stomach when participating in the care of a patient whom the student felt did not want him there but who still gave consent to him being there. The feeling of unease disappeared as students became more accustomed to the situation and performing examinations. One student expressed that the knot in his stomach was a product of expectations.

"The first week, I had a feeling of having a knot in my stomach every time I met a patient if I was present, but they didn't want me there but failed to say so…Then you get used to it, and it became much better, and at the end of the clinical clerkship, the feeling is totally different. Now it feels more natural to be present, and now you are in control of the situation, and that feels good." - Male, 28.

## Discussion

The majority of the students who participated in this study stated that they felt confident performing pelvic examinations (speculum and/or vaginal examinations) at the end of their clinical ObGyn clerkship. The FGD participants expressed that they felt more comfortable performing pelvic examinations than other types of examinations. Since we used the term pelvic examinations for both speculum and vaginal examinations, we cannot make an exact comparison with the results reported in previous studies that treated these concepts separately. However, the mean number of examinations performed per student was higher in the current study than in previous studies.^4^ The students expressed in the FGD that the only way to properly learn clinical examinations is by performing them many times.

### The influence of gender

The majority of students stated feeling encouraged to participate in the clinic during the gynaecology rotation, with no difference with respect to gender. The positive experience of feeling encouraged by the supervisor when performing examinations and the feeling of being included as part of the team was equally common among female and male participants. However, male students more frequently experienced being denied permission to participate in various tasks. In agreement with previous studies, a significant difference was observed in being denied permission to observe or/and perform examinations between male and female students.^5^^,^^16^

More female than male students experienced that their gender influenced their clinical clerkship. All women who experienced this influence reported that it was positive (increased their opportunities to participate). In contrast, male students who experienced a gender influence stated that this influence was negative. A similar but less pronounced trend was reported regarding the obstetrics rotation. The observation of how gender impacts the clerkship experience is concordant with previous studies.^4^^,^^5^^,^^17^

There was no difference with respect to gender in the experience of discrimination or harassment by a doctor, midwife or auxiliary nurse in the gynaecology department or the delivery ward.

At the end of the clerkship, 130/160 students stated that their interest in ObGyn had been influenced by their clerkship experiences, and the majority, with no significant difference in respect to gender, reported that their interest in the speciality had increased. This differs from previous studies, where a more pronounced increase in interest was seen among male than female students at the end of the clerkship.^5^^,^^6^ When asked about the possible need to prepare for the clinical rotation, a majority of students stated that they did not see any value in preparation. In this study, there was no difference with respect to gender.

### The role of the supervisor

A concern that was stressed in the FGD was the absence of opportunities to discuss patients' conditions and receive feedback from the supervisor without the patient being within hearing distance. A feeling of uneasiness was reported about having to expose one's inadequate knowledge by asking questions regarding the findings and the patient's condition with the patient still present in the room. Perhaps this could explain why some students did not feel confident performing pelvic examinations at the end of their clerkship since they had found it difficult to interpret their findings when performing an examination without the opportunity to discuss them with the supervisor. Previous studies have shown that the encouragement of discussion and reflection increases learning opportunities for medical students; thus, it is important to allow time and provide structure for these activities so that they can be integrated into the learning and assessment system.^22^

Most of the participants expressed a preference for the clinical tutors who explained step by step what they were doing while performing examinations and who included the student in decisions, adjusting the information given to the knowledge level of the student. This attitude also made the student feel more a part of the team. Engaging the student by explaining procedures step by step also makes the patient feel more at ease and less vulnerable when being examined.8 One participant expressed that the more engaged the clinical tutor was with him, the more engaged he was in learning. If the supervisors led the patients to see the students as competent and professional by introducing them as future colleagues, the students experienced that the patients were more ready to accept the presence of students. The students also experienced it as easier and more natural to participate in the care of the patients when they had been introduced early in the process and were more at ease with the situation when they felt that their presence was appreciated by both the clinical tutors and the patient.

The majority of the students reported no experience of being discriminated against during the ObGyn clerkship. When this happened, it was more likely to be by a doctor. This may be because doctors more frequently serve as clinical tutors in gynaecology. In line with this, during the obstetrics clerkship, when discrimination was reported, it was likely to be perpetrated by midwives. In the FGDs, there were no mentions of discrimination. It was discussed that some clinical tutors were more engaged with their students than others and that this positively influenced the tendency among patients to allow participation.

### Patient autonomy

In the focus group discussions, several of the students expressed that they were aware of and sympathetic to the possibility that they could be denied permission by the patient to participate in observing and performing examinations, especially if the student was male. Acceptance of patients' autonomy and right to deny student participation has also been demonstrated in a previous study.^4^ Nevertheless, even if most students were aware of and accepted the risk of not being granted permission, the need for a reason for not being allowed to participate was expressed. Otherwise, it was felt that there could be a risk that one would begin to blame and question oneself and question the supervisor's agenda.

Some students expressed that they had based their negative expectations to a large extent on stories from senior students who had experienced being denied permission to participate, mainly because of their male gender. In previous studies, it has been noted that male students may hold preconceived biases against ObGyn before their clerkship, and they express a higher frequency of anxiety than female students about performing vaginal examinations.^4^^,^^5^ There was a trend among the participants of being pleasantly surprised by their clerkship experience and feeling surprised about being allowed to participate. In the FGD, it was brought to light that misgivings and stories from senior students might emerge because people have a greater tendency to share negative experiences than positive experiences. Therefore, negative experiences should be followed up and addressed by clinical tutors to avoid negative expectations being transferred to future students.

### The use of professional patients

In the FGDs, the students expressed appreciation for having been offered the opportunity to perform their first pelvic examination on a PP. Since PPs are not used in all medical schools in Sweden, we did not aim to investigate the relationship between the use of a PP and confidence in performing examinations at the end of the ObGyn clerkship. However, the PP experience was highlighted in the FGDs as an important part of learning to perform examinations. This accords with previous studies that have shown that the use of PPs helped students learn how to handle their clinical patients.^20^^-^^22^ Furthermore, students who were instructed by PPs performed better on pelvic examinations, expressed more confidence in performing examinations and demonstrated better communications skills than those who had not been instructed by a PP.^20^^-^^23^

### Strengths and limitations

The mixed-methods design allowed a more in-depth investigation of the topic. The survey provided an overview of how medical students in Sweden experience their ObGyn clerkship and allowed us to explore whether the results presented in previous studies applied to the context of Swedish medical schools. Through complementary FGDs, we obtained a more profound understanding of students' perceptions.^12^ A challenge in conventional content analysis is failing to develop a complete understanding of the context. We chose to establish credibility through the use of systematic text condensation and complementarity to ensure the validity of the results.^14^^,^^15^

The methodology used to invite participants allowed us to reach students at several universities while keeping responses confidential. A total of 160 students were included, which is more than in previous similar studies.^4^^,^^5^ All questions were mandatory; thus, there were no missing data. Possible limitations of this approach were the lack of control over how many students were reached by invitation to participate and over the gender distribution. The gender distribution is, however similar to what is described in Swedish Medical schools, where it is estimated that about 55% of the students are female. Received the study invitation at different universities may have contributed to the varying number of recruited participants. Although we based our survey on a previously used and piloted survey, there may still be a risk that some questions were hard to interpret, understand or answer, which may have affected the results.

In this study, two FGDs were conducted, both including male and female students. Since a gender difference was demonstrated in the results, it may be of interest for further research to conduct FGDs, including both homogenous and mixed groups, to investigate whether new phenomena can be described. Clearly, group discussion can enhance but also obstruct the mediation of experiences depending on group dynamics and interaction. In the FGDs conducted in this study, all participants took an equal part in the conversation, and no gender differences could be seen in the tendency to dominate the discussion. This was monitored by both the observer and moderator. It could also be mentioned as a strength that there was no compensation offered to the participants.

## Conclusions

The vast majority of medical students reported a positive learning experience in performing pelvic examinations (speculum and/or vaginal) during the ObGyn clerkship. The clinical tutor had a fundamental role in providing learning opportunities by including the student in patient care activities. There was a significant gender difference with respect to negative expectations and whether students were allowed to participate; however, no difference was observed regarding students' confidence in performing gynaecological examinations at the end of the clerkship. The most important factor identified by the students where the use of PPs when performing their first pelvic examination. However, much less is known about the role of the tutor and the use of PPs for medical students when training other intimate examinations. In conclusion, most students report a positive and adequate learning experience and factors to improve further ObGyn clerkship may be using PPs and highlighting the important role of the tutor when introducing the student to the patient. Similar investigations on students experience in training other intimate examinations could be considered to improve the learning process in these situations.

### Acknowledgments

We are grateful to all the students who participated and the teachers and coordinators who helped with the recruitment.

### Conflict of Interest

The authors declare that they have no conflict of interest.
